# Physicochemical parameters - hydration performance relationship of the new endodontic cement MTA Repair HP

**DOI:** 10.4317/jced.56013

**Published:** 2019-08-01

**Authors:** María del Carmen Jiménez-Sánchez, Juan-José Segura-Egea, Aránzazu Díaz-Cuenca

**Affiliations:** 1 Department of Stomatology, Faculty of Dentistry, University of Sevilla, Sevilla, Spain; 2 Materials Science Institute of Sevilla (ICMS), Joint CSIC-University of Sevilla Center, Sevilla, Spain; 3 Networking Research Center on Bioengineering, Biomaterials and Nanomedicine (CIBER-BBN), Spain

## Abstract

**Background:**

To characterize the chemical composition and textural parameters of the MTA Repair HP precursor powder and their influence to hydration performance.

**Material and Methods:**

Un-hydrated precursor material was characterized by X-ray diffraction (XRD), Fourier transform infrared spectroscopy (FT-IR), X-ray fluorescence (XRF), laser diffraction (LD), N2 physisorption and field emission gun scanning electron microscopy (FEG-SEM). Setting time was assessed according to ASTM specification C 266. Hydrated material was analysed by XRD, FT-IR, energy dispersive X-ray (EDX) analysis and FEG-SEM.

**Results:**

Ca3SiO5 and Ca2SiO4, in addition to CaWO4 as radiopacifier are the main compositional phases. Other measured parameter indicates high specific surface area of 4.8 m2 g-1, high aluminium content of 1.7 wt.% and low initial and final setting times of 12 and 199 min, respectively. Singular microstructural features consisting of high aspect ratio nanoparticles are main constituents of un-hydrated precursor. Besides, FEM-SEM observation shows notably growth of hexagonal shaped plate-like morphologies homogeneously distributed along the sample during hydration process.

**Conclusions:**

The short setting time measured for HP Repair, is correlated with high surface area of precursor powder, high Al content and the absence of compositional sulphate phases.

** Key words:**Bioactive endodontic cements, hydration performance, MTA HP Repair, physicochemical parameters.

## Introduction

Mineral Trioxide Aggregate (MTA) endodontic cement, is a versatile material indicated for clinical use as root-end filling material, perforation repair, vital pulp therapy or apical barrier formation in teeth with open apexes (1). Since the first clinical approved formulation, ProRoot MTA (2), very intensive research is performed to find new formulations with improved properties in terms of hydration process and kinetics (3-5) biomechanical performance (6,7) and the role played by the radiopacifying agents (8,9).

MTA main compositional formulation is based on calcium silicates in the form of tricalcium (Ca3SiO5) and dicalcium (Ca2SiO4) silicate. Additionally, a radiopacifying material as bismuth, zirconia, tantalum or tungsten oxide (10,11) is other important component. The powder ProRoot MTA contains bismuth oxide as radiopacifier, which plays a main role in cement hydration and in tooth discoloration (12). Therefore, new cements containing alternative radiopacifiers elements to replace bismuth have been prepared (9,13). Furthermore, other modifications, including variations of cement formulation (10,14) and the introduction of additives (6,7,15), have also been made to overcome disadvantages such as long setting time, high cost and difficult handling characteristics. The alteration of the composition of the material modifies its physical-chemical and functional properties, changing its biomechanical behaviour and its bioactive response.

In this context, a new material, MTA Repair HP (Angelus, Londrina, Brasil), with reported great plasticity and handling (11), in which the radiopacifier bismuth oxide has been replaced by calcium tungstate (CaWO4), has come onto the market. In this study, the chemical composition, textural properties and microstructure of the precursor ceramic powder of MTA Repair HP is characterised and analysed in relation with its hydration performance and setting time.

## Material and Methods

MTA Repair HP (Angelus, Londrina, Brasil) was used in this study.

MTA Repair HP powder material characterization: X-ray diffraction (XRD) analysis was performed with a PANalytical X’Pert PRO diffractometer (Almelo, Netherlands), using Cu-Kα radiation (0.154187 nm). The difractometer was operated at 45 kV and 40 mA using a step size of 0.02 and a 500 s exposure time. Phase identification was accomplished by use of search-match software utilizing ICDD database (2002, Pennsylvania, USA). Fourier transform infrared (FT-IR) spectra were collected in transmission configuration in the 4000-400 cm-1 range using 4 cm-1 interval in Nicolet IS50 FT-IR of Thermo Scientific (Madison WI, USA). The compositional analysis was performed by X-ray fluorescence (XRF) using the sequential Spectrometer AXIOS WD-XRF (PANalytical, Malvern, UK). The particle size distribution of the powder was measured in a Malvern Sizer laser diffraction (LD) instrument (Southborough, MA, USA), using an active beam length of 2.4 mm and a 300-RF lens. Likewise, textural parameters were determined by N2 physisorption. Adsorption–desorption isotherms were collected on a Micromeritics Tristar 3020 gas adsorption analyzer (Norcross, GA 30093-2901, USA). The specific surface area was determined by the BET (Brunauer-Emmett-Teller) method (16) after degassing at 523 K for 2 h in a nitrogen stream. Total pore volume was obtained from the N2 amount adsorbed at 0.99 relative pressures. The microstructure of the material was studied by field emission gun scanning electron microscopy (FEG-SEM) using a HITACHI S-4800 (Tokyo, Japan). Images were recorded at an accelerating voltage of 2 kV. Energy dispersive X-ray (EDX) analysis was carried out at 10 kV with an EDX Bruker XFlash 4010 detector.

Hydrated MTA Repair HP process and analyses: The powder was mixed according to manufacturer’s instructions with Milli-Q water only, to avoid the influence of specific product manufacturer additives, and to analyse then specific ceramic powder compositional properties. The manual mixing was performed adding the liquid to the powder on a glass slab, and the cement was blended using a metal spatula. A paste with homogeneous consistency was obtained. The paste was compacted in a silicone mould of 10 mm in diameter and 4 mm high. Three silicone moulds were filled and stored in an incubator at 37oC and 95% relative humidity. The setting time was determined according to American Society for Testing and Materials specification C 266. 113.4±0.5 and 453.6±0.5 Gillmore needles were used respectively to determine the initial and the final setting times. This procedure was repeated at 60-second intervals, and the time was measured using a digital chronometer. The setting times were measured from the start of mixing to the time at which no indentations could be seen on the surface of the specimen. Measurements were performed 3 times. XRD, FT-IR, FEG-SEM and EDX analyses of hydrated material were performed using the same equipment and parameters as detailed above for Repair HP powder precursor characterisation.

## Results

-Un-hydrated ceramic powder analyses

**Figure 1:**
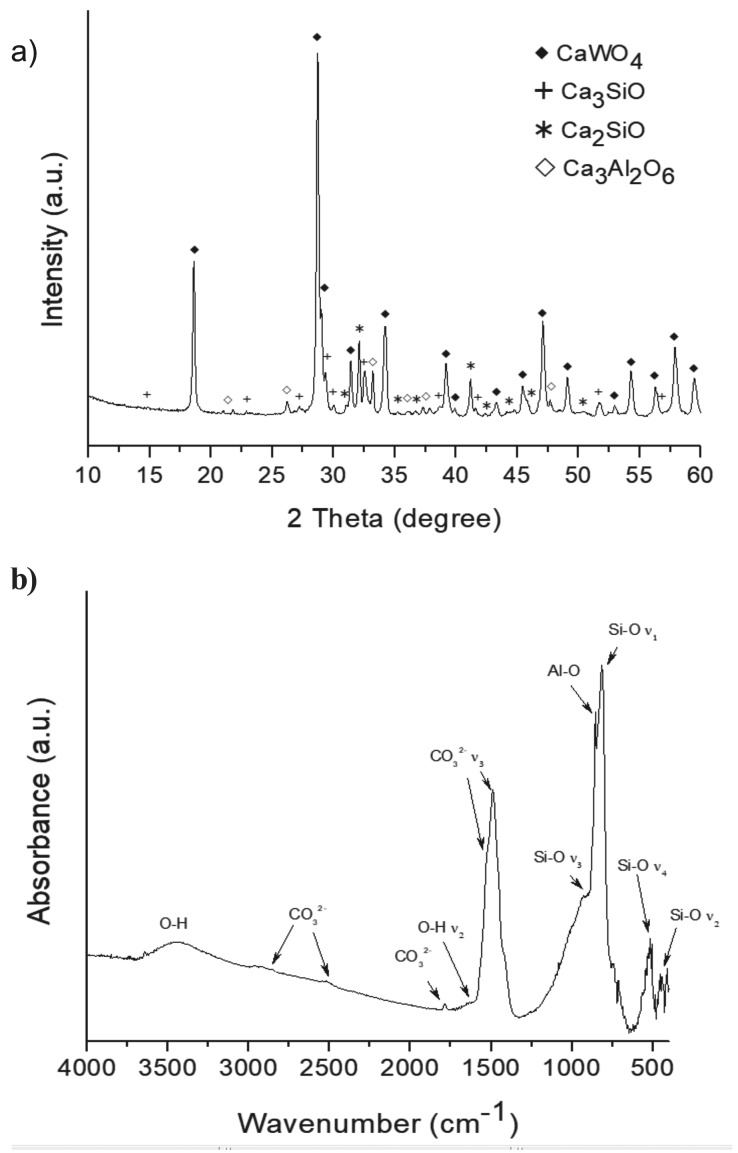
a) X-ray diffraction (XRD) and, b) FT-IR analyses of un-hydrated MTA Repair HP material.

MTA Repair HP composition specification supplied by the manufacturer is listed in  Table 1. Together with radiopacifier calcium tungstate, CaWO4 (PDF 01-077-2233), our XRD analysis (Fig. 1a) confirms tricalcium silicate, Ca3SiO5 (PDF 01-086-0402), and dicalcium silicate, Ca2SiO4 (PDF 01-077-0409), as major components. Infrared spectrum (Fig. 1b) shows likewise, Si–O asymmetric stretching (ѵ3), and Si–O bending (ѵ4 and ѵ2) centred respectively at 925, 522, and 452 cm−1 of the calcium silicate components (17). Besides, XRD shows the presence of tricalcium aluminate, Ca3Al2O6 (PDF 00-032-0148), and FT-IR confirms high absorbance of Al-O bonds (Fig. 1b).

**Table 1:**
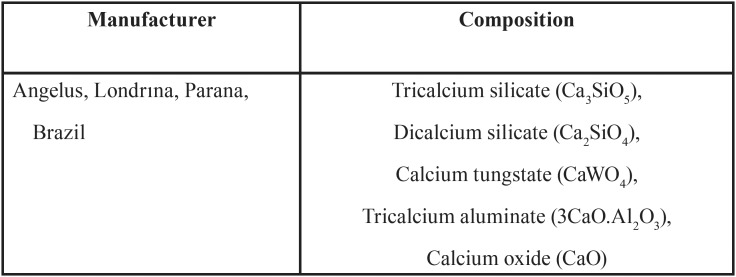
MTA Repair HP manufacturer chemical composition Specifications.

**Figure 2:**
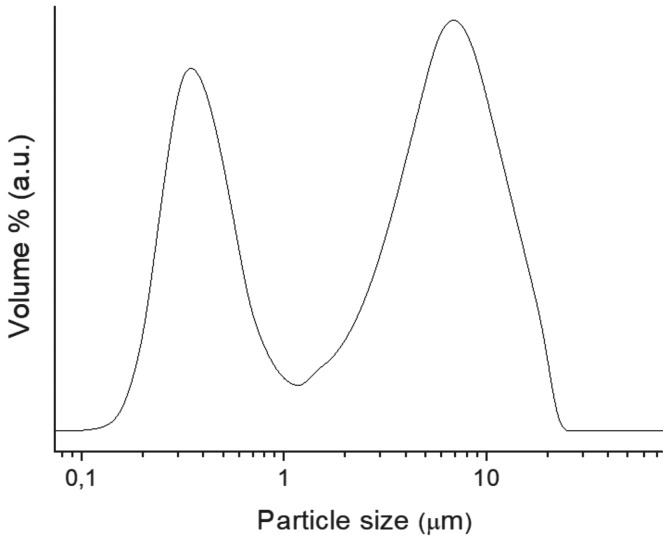
Particle size distribution of un-hydrated MTA HP Repair material.

**Figure 3:**
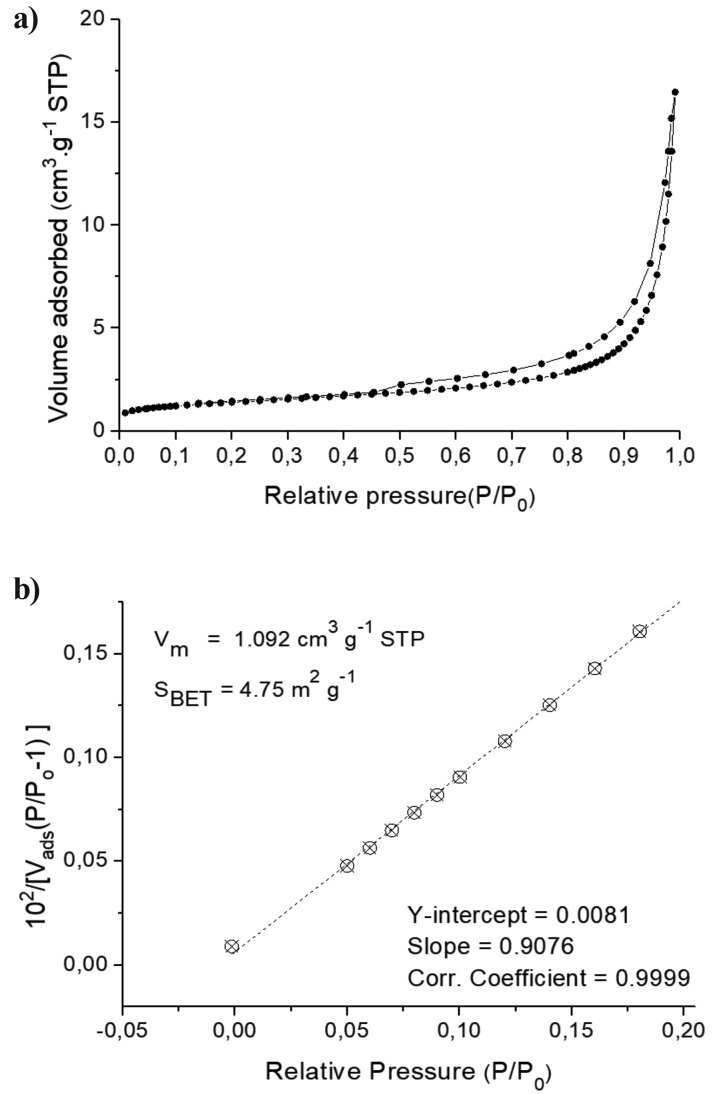
N2 physisorption analysis of un-hydrated MTA HP Repair material: a) Adsorption-desorption isotherm; b) BET method analysis using the 0.0-0.2 P/Po adsorption branch range. Vm: Monolayer capacity volume.

**Figure 4:**
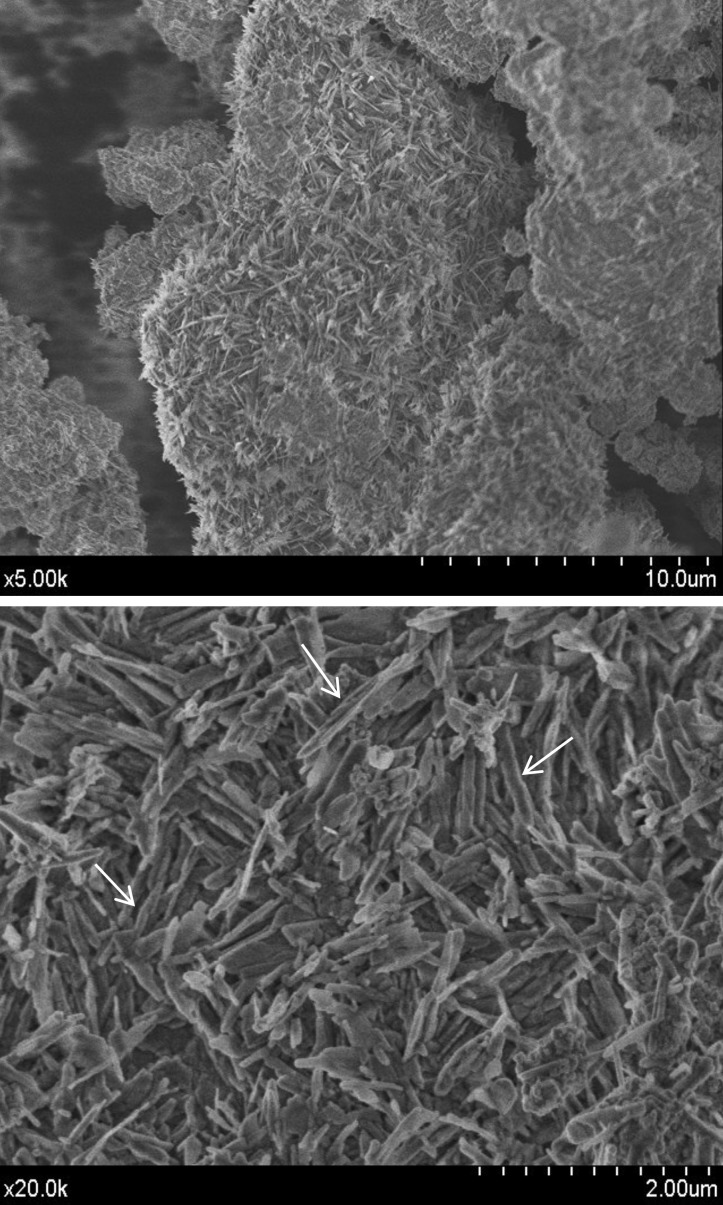
FEG-SEM secondary electron micrographs at two different magnifications of un-hydrated MTA Repair HP material exhibiting a finer microstructure of elongated nanometric features in thickness (white arrows drawn perpendicular to larger axis).

A quantitative analysis of the composition was carried out using XRF as presented in  Table 2. In good agreement with XRD and FT-IR analysis, the precursor powder show high Ca, W, Si and Al content, with some other minoritary components as Sr, Mg and Na, which are present, bellow XRD detection limits. The particle size measurements are displayed in Figure 2. A bimodal distribution with two maxima at 0.3 and 6.9 μm is observed. Physisorption analysis is displayed in Figure 3. Type IIb adsorption-desorption isotherms (Fig. 3a) exhibit Type H3 hysteresis (18). Specific surface area calculated by BET (Fig. 3b) is 4.8 ± 0.0 m2g-1 being the total pore volume 0.015 ± 0.001 cm3 g−1. The study of the microstructure by FEG-SEM (Fig. 4) showed the resolution of homogeneous small needle-like morphologies of 50-100 nm sizes thickness over the entire surface of the material.

-Hydrated ceramic MTA HP Repair analyses

**Table 2:**
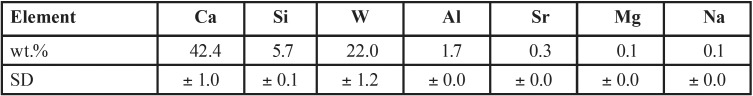
Quantitative analysis by X-ray fluorescence (XRF) of MTA Repair HP powder precursor material.

**Figure 5:**
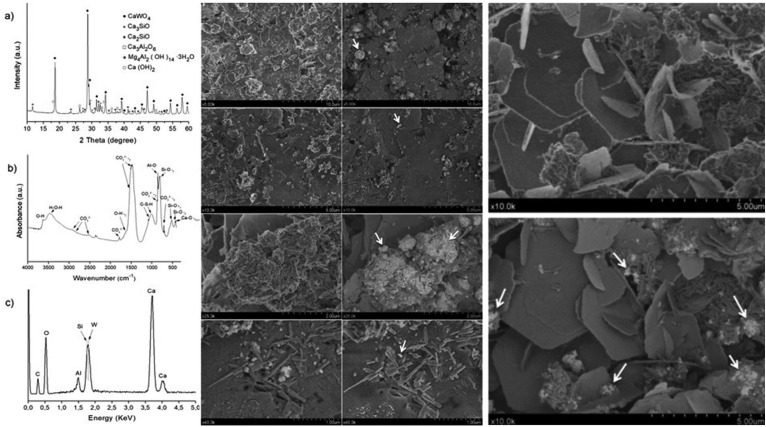
LEFT. Characterization of hydrated MTA HP Repair material: a) XRD pattern; (b) FT-IR and, (c) EDX analysis. CENTRE. Secondary (left column) and back-scatter (right column) FEG-SEM images of hydrated MTA HP Repair material surface at four different magnifications. Scale bars: 10 μm; 5 μm; 2 μm and 1μm. White arrows drawn indicate CaWO4 component distribution. RIGHT. Comparison of FEG-SEM second (top) and back-scatter (down) images of the hydrated MTA Repaid HP material surface. White arrows drawn indicate CaWO4 phase distribution.

After addition of water, MTA Repair HP components react producing calcium silicate hydrate (CSH) and calcium hydroxide Ca(OH)2. XRD analysis is displayed in Figure 5 left, a. A shoulder signal at 2Ɵ = 18.0º identified Ca(OH)2 (PDF 00-044-1481), is also merging to CaWO4 peak at 2Ɵ = 18.6º. Clearly, a small angle peak at 2Ɵ = 11.7º and 23.5º matched with Mg4Al2(OH)143H2O (PDF 00-035-0964). The results of infrared spectroscopy are shown in Figure 5, left, b. The material showed a sharp peak at about 420 cm-1 that can be assigned to the lattice vibration (Ca-O) of hydrated tricalcium aluminate19. The three bands at 714, 875 and 855 cm-1, 1490-1420 cm-1 and 2950-2500 cm-1 corresponded to carbonate build up from the reactions of atmospheric CO2 with calcium hydroxide (17,20). Symmetric and asymmetric stretching vibrations of O-H adsorbed water molecules caused the broad band centred at 3400 cm-1. The sharp band at 3640 cm-1 corresponded to the OH band from Ca(OH)2 (21). Secondary and back-scatter electron images of the hydrated MTA HP Repair surface (Fig. 5, centre and right) showed aggregate particles formed as clusters. A flat background was noticed, breaking off by big bumps where particles with platelet morphology were visible (Fig. 5, right). Radiopacifying CaWO4 provoked bright features, marked with arrows at the micrographs. The initial and final setting times measured were 12 ± 2 min and 199 ± 5 min, respectively.

## Discussion

The results of the present study demonstrate that the main chemical composition of MTA Repair HP consist of calcium silicates in the form of Ca3SiO5 and Ca2SiO4, in addition to CaWO4 as radiopacifier element. Major by-product phases produced during the hydration reaction, leading to the setting and hardening of the cement, were calcium silicate hydrate (CSH) and calcium hydroxide Ca(OH)2. The absence of XRD peak corresponding to the major hydrated product, CSH, can be explained by the poorly crystallised calcium silicate formation, together with the nanoscale crystalline structure of CSH, causing to appear amorphous in XRD (4,22). The peak at 2Ɵ = 18.0º is used to identify the production of Ca(OH)2 (4), in this case, overlapping a CaWO4 peak (Fig. 5a). New peaks at 11.7º and 23.5º matched with Mg4Al2(OH)143H2O phase, which may suggest that aluminium is involved in the hydration process. FT-IR analysis showed bands within the 1011-1080 cm-1 range, confirming the formation of CSH polymerized silica (21). Powder precursor (Fig. 1b) shows an intense Si-O (ѵ1) band before hydration which is indicative of [SiO4]4- tetrahedron lower ordered structure. After hydration, Al-O band intensity increases relative to Si-O (ѵ1), highlighting Al as a hydration active element. Accordingly, hydration of Ca3Al2O6 phase results in new XRD peaks corresponding to Mg4Al2(OH)143H2O. The presence of aluminium is also confirmed by EDX after hydration (Fig. 5c). However, XRF analysis indicates very low content of 0.1wt. % magnesium which brings us to hypothesised that divalent calcium could substitute magnesium in a Mg4Al2(OH)143H2O related phase.

Short setting times measured for MTA Repair HP, could well be promoted by the high surface area of the material (4.8 m2 g-1). Previous reported data for other commercial MTA materials are significantly lower: 1.0 m2 g-1 for ProRoot (3) and MTA Angelus (14); and 1.5 m2 g-1 for MTA Plus (3). In this work, the increase of the surface area is correlated with FEG-SEM resolution of high aspect ratio nanometric particles. The higher surface area correlates with smaller particle sizes which greatly faster the setting times (23). In addition, the low amount of sulphate, confirmed by XRF, is correlated with shorter setting times as calcium sulphates are setting regulators to avoid a rapid desiccation of powder precursor paste (19). Besides, FEG-SEM observations of un-hydrated precursor indicate a fine microstructure consisting of submicron-elongated features of nanometric thickness in good agreement with Type IIb isotherm results which is characteristic with aggregates of plate-like particles.

FEG-SEM observations of hydrated material (Fig. 5, centre), show “Hadley-like” grains (hollow-shells) and gaps likely between remnants cement cores and shell of hydration reaction products (24). Furthermore, similar hexagonal plate-shaped crystals on the surface (shown in Fig. 5, right) has been associated to Ca(OH)2 formation and, facetted crystals growth to CaCO3 derived from carbonation of Ca(OH)2 (4). However, flat CSH sheet formation from hydrated SiO4 monomer crystallisation in a two-dimensional direction cannot be disregarded (4).

## Conclusions

Physicochemical characterization of MTA Repair HP shows CaWO4, Ca3SiO5 and Ca2SiO4 as main compositional phases. Also, XRF quantified aluminium content of 1.7 wt.% is in good agreement with XRD detection of Ca3Al2O6 phase. The short setting time measured for HP Repair is correlated with precursor powder high surface area of 4.8 m2 g-1, high Al content and the absence of compositional sulphate phases. Besides, FEG-SEM observation indicates high aspect ratio nanoparticles final form fabrication of un-hydrated powder material.
